# Pomegranate: Its Health and Biomedical Potential

**DOI:** 10.1155/2013/903457

**Published:** 2013-10-28

**Authors:** Ari M. Mackler, David Heber, Edwin L. Cooper

**Affiliations:** ^1^Clinical Development, POM Wonderful, Los Angeles, CA 90064-1549, USA; ^2^Division of Clinical Nutrition, David Geffen School of Medicine at UCLA, University of California, Los Angeles, CA 90095-1742, USA; ^3^David Geffen School of Medicine at UCLA, University of California, Los Angeles, CA 90095-1763, USA

Diet and lifestyle have long been recognized as important modifiers of health status. Modern scientific methods are being used to uncover, demystify, and validate the benefits of dietary constituents in health promotion. Likewise, many different foods are currently being studied for their role in the management of numerous disorders and conditions ranging from metabolic and inflammatory disorders to cardiovascular disease and cancer. Indeed, our best medicines may ultimately turn out to be botanicals, sourced from the 150,000 to 200,000 edible plant species on earth. These compounds are simply awaiting discovery and development. The concept of functional foods is evolving with insights into the mechanisms of action of a number of bioactive compounds found in foods. In this regard, this special issue focuses its attention on the emerging science of the pomegranate and its rich store of bioactive phytochemicals.

The pomegranate's ability to combat oxidative stress, augment the activity of nitric oxide, and modulate inflammatory pathways serves as the scientific basis for much of its recently discovered health benefits. As an example, a balance between free radicals and antioxidants is necessary for proper physiological function. If free radicals overwhelm the body's ability to regulate them, a condition known as oxidative stress ensues. Downstream, free radicals adversely alter lipid, protein, and DNA within cells, contributing to the etiology of a number of common chronic diseases and conditions associated with aging. The health benefits of pomegranate juice and extracts is derived from a spectrum of bioactive agents. In addition to the unique family of polyphenols in the pomegranate called punicalagins, pomegranate phytochemicals include anthocyanins, flavanols, and a seed oil which can be converted to conjugated linoleic acid. Among fruits, the hydrolysable tannin called punicalagin is uniquely found in the pomegranate. This is the largest polyphenol antioxidant with a molecular weight of over 1000 Daltons. These large molecules are hydrolyzed in the intestine and release ellagic acid into the bloodstream. Punicalagin, punicalin, gallagic acid, and ellagic acid account for the majority of the ellagitannins. The gut bacteria convert these ellagitannins into urolithins which are small molecules with antioxidant and other biological effects. Interestingly, the relevance of newly identified compounds to the mechanisms of action underlying the benefits of pomegranate juice and extracts has become an important path for future exploration and scientific investigation. To this end, purified extracts have proven to be valuable tools to explore and understand mechanisms of action of pomegranate juice.

The pomegranate occupies a prominent place in art, religious symbolism, and traditional medicine dating back thousands of years. A look into our modern literature will point to research from as far back as 1821. Rigorous investigation, however, only began some 20 years ago. Within this special issue both original research and review articles, covering both mechanism of action and clinical investigations, are included. From a mechanism perspective, articles in this issue evaluate the biological effects of urolithins and assess the role of these gut-produced metabolites in potential mechanism-of-action schemas for pomegranate activity. Additionally, authors describe work with cancer models that are helping to identify subcellular molecular mechanisms of action, such as the *β*-Catenin Signaling Pathway. From a clinical perspective, authors have prepared analyses on such varied clinically relevant topics as cardiovascular health, memory loss, immunity, and cancer. As described by these investigators, although pomegranate demonstrates promise across different patient types, the underlying mechanisms of action are often similar (e.g., protecting against the damaging effects of hypoxia).

It is our hope that this compilation of data will advance our knowledge of this ancient fruit's chemistry and further its application to the most perplexing chronic diseases facing mankind today.

*Ari M. Mackler* *Ari M. Mackler* *David Heber* *David Heber* *Edwin L. Cooper* *Edwin L. Cooper*

